# The Multiple Myeloma Landscape: Epigenetics and Non-Coding RNAs

**DOI:** 10.3390/cancers14102348

**Published:** 2022-05-10

**Authors:** Isabel F. Coira, Rafael Rincón, Muriel Cuendet

**Affiliations:** 1 School of Pharmaceutical Sciences, University of Geneva, 1211 Geneva, Switzerland; isabel.fernandezcoira@unige.ch (I.F.C.); rafael.rincon@unige.ch (R.R.); 2 Institute of Pharmaceutical Sciences of Western Switzerland, University of Geneva, 1211 Geneva, Switzerland

**Keywords:** CRISPR-Cas, long non-coding RNA, microRNA, multiple myeloma, non-coding RNA

## Abstract

**Simple Summary:**

Recent findings in multiple myeloma have led to therapies which have improved patient life quality and expectancy. However, frequent relapse and drug resistance emphasize the need for more efficient therapeutic approaches. The discovery of non-coding RNAs as key actors in multiple myeloma has broadened the molecular landscape of this disease, together with classical epigenetic factors such as methylation and acetylation. microRNAs and long non-coding RNAs comprise the majority of the described non-coding RNAs dysregulated in multiple myeloma, while circular RNAs are recently emerging as promising molecular targets. This review provides a comprehensive overview of the most recent knowledge on this topic and suggests new therapeutic strategies.

**Abstract:**

Despite advances in available treatments, multiple myeloma (MM) remains an incurable disease and represents a challenge in oncohematology. New insights into epigenetic factors contributing to MM development and progression have improved the knowledge surrounding its molecular basis. Beyond classical epigenetic factors, including methylation and acetylation, recent genome analyses have unveiled the importance of non-coding RNAs in MM pathogenesis. Non-coding RNAs have become of interest, as their dysregulation opens the door to new therapeutic approaches. The discovery, in the past years, of molecular techniques, such as CRISPR-Cas, has led to innovative therapies with potential benefits to achieve a better outcome for MM patients. This review summarizes the current knowledge on epigenetics and non-coding RNAs in MM pathogenesis.

## 1. Introduction

Multiple myeloma (MM) accounts for about 10% of hematological malignancies. It is a plasma cell malignancy that originates from the post-germinal lymphoid B-cell lineage, and is characterized by an uncontrolled clonal growth of plasma cells. It is preceded by monoclonal gammopathy of undetermined significance (MGUS) that progresses to smoldering myeloma and finally to symptomatic MM [1].

Frequently, these clones of plasma cells invade the adjacent bone and occasionally infiltrate multiple organs, causing symptoms such as hypercalcemia, renal insufficiency, anemia, and bone lesions. In the past decades, the therapeutic landscape of MM has improved with the development of targeted therapies, chemotherapeutic agents, and immunotherapy. Despite this, relapses are common [2]. A better understanding of mechanisms involved in MM is necessary to advance the development of new treatments. This review aimed to summarize the epigenetic factors and non-coding RNAs (ncRNAs) involved in MM, as well as to discuss the therapeutic possibilities they can offer. Classical epigenetic factors, such as methylation and acetylation, are first introduced. This is then followed by ncRNAs, whose importance in cancer has increased recently. To conclude, current therapeutic approaches and future perspectives are discussed.

## 2. Methylation

DNA methylation is a central epigenetic modification in cancer. It plays an important regulatory role in transcription, chromatin structure and genomic stability, X chromosome inactivation, genomic imprinting, and carcinogenesis [3]. Global hypomethylation in cancer cells was one of the first epigenetic alterations found in carcinogenesis. Moreover, certain genes are inactivated due to hypermethylation of CpG islands in regulatory regions. This process is catalyzed by DNA methyltransferases (DNMT) and involves the addition of a methyl group to the carbon 5 position of the cytosine ring in the CpG dinucleotide, generating a 5-methylcytosine (5mC) [4]. The opposite process of demethylation is mainly catalyzed by TET enzymes, which can oxidize 5mC to 5-hydroxymethylcytosine (5hmC), 5-formylcytosine (5fC), and 5-carboxylcytosine (5caC). These oxidized products can then be removed by base excision repair and substituted by cytosine in a locus-specific manner [5]. However, despite the finding of TET2 loss-of-function mutations in some hematological malignancies, there is very few knowledge about their role in MM [6].

Methylation patterns have been shown to be different depending on the stage of MM progression. In non-malignant stages and MGUS, demethylation occurs mainly in CpG islands. At the transition from MGUS to MM, the key feature is a strong loss of methylation, associated with genome instability. In malignant stages, changes in methylation are widespread in the genome, outside of CpG islands, and affect various pathways, such as cell cycle and transcriptional activity regulators [7]. *DNMT3A* is hypermethylated and underexpressed in MM, leading to a global hypomethylation. Interestingly, DNA hypermethylation in B-cell specific enhancers seems to be a key feature of MM-staged cells. These hypermethylated regions are located in binding sites of B-cell specific transcription factors, thus leading to an impaired expression of those and, consequently, a more non-differentiated cell profile in MM cells. This hypermethylation in B-cell-specific enhancers has been found in stem cells; it is progressively eliminated in non-malignant B cells and reacquired again in MM cells [8].

Genomic studies have been performed to explain the role of promoter hypermethylation of tumor suppressor genes. Preliminary studies revealed that in MM patients, there was aberrant methylation in genes such as *SOCS-1*, p16, *CDH1*, *DAPK1*, and p73. Hypermethylation of crucial tumor modulating genes, such as *GPX3*, *RBP1*, *SPARC*, and *TGFBI* has been associated with a significantly shorter overall survival, independently of age, International Staging System (ISS) score, and adverse cytogenetics [9,10].

Moreover, several signaling pathways were found to be dysregulated in MM. STAT3 overexpression due to promoter hypermethylation was associated with an adverse prognosis and was mainly induced by IL-6 signaling [11]. DNA methyltransferase inhibitors (DNMTi), such as 5-azacytidine, were shown to revert hypermethylation and exerted synergistic anti-MM effects with bortezomib [12]. Therefore, several clinical trials have been conducted to assess DNMTi efficacy in combination with anti-MM agents, such as lenalidomide or dexamethasone [13].

Beyond DNA, histones are also found to undergo methylation in the context of MM, especially due to the action of the histone methyltransferases MMSET and EZH2. The chromosomal translocation t(4;14) leads to an overexpression of MMSET in 15–20% of MM cases [14]. MMSET—also known as NSD2—is capable of dimethylating histone H3 on its lysine 36 (H3K36me2). This epigenetic marker is mainly seen in actively transcribed genes, suggesting that MMSET is a central epigenetic player in MM by activating several oncogenic genes [15,16]. Its wide repertoire of oncogenic downstream targets such as NF-κB, Bcl-2, SLAMF7, or cyclin E2 suggests that MMSET inhibition could lead to anti-cancer effects in MM, via small molecules or siRNA delivery [17]. The methyltransferase EZH2 is a catalytic subunit of the polycomb repressive complex 2 which catalyzes the trimethylation of histone H3 on its lysine 27 (H3K27me3). Contrarily to the demethylation on lysine 36 induced by MMSET (H3K36me2), this methylation feature is linked to gene repression. Therefore, *EZH2* acts as an oncogene by blocking different tumor suppressor genes, which strictly regulate the cell cycle or cell differentiation [18]. The oncogenic role of *EZH2* is not exclusive to MM, and it is associated with poor prognosis in many other malignancies [19]. It also takes part in drug resistance in some contexts, as its phosphorylation and consequent inactivation can promote the expression of antiapoptotic genes. This phosphorylation has been observed to be induced by the contact with stromal cells, suggesting this interaction as one of the mechanisms in MM resistance [20].

## 3. Acetylation

Acetylation is one of the major reversible post-translational modifications that introduces an acetyl group on histone lysine residues, thus modifying the gene expression pattern. It involves a dynamic process, consisting of a balance between the activity of histone acetyltransferases (HATs) and histone deacetylases (HDACs). This balance serves as a key regulator that influences many cellular processes such as cell cycle, chromatin structure, and gene expression [4].

HATs catalyze the attachment of acetyl groups, resulting in a less condensed chromatin structure. CREB-binding protein CBP/p300 family is a HAT type A enzyme, whose mutations are often related to cancer development. It is located in the nucleus and involved in the acetylation of histones. CBP/p300 is dysregulated in hematological malignancies [21] and, in the case of MM, inhibition of CBP/p300 has been shown to induce cell death via the reduction of IRF4 expression [22]. This could open a promising therapeutic strategy but however, the majority of studies are focused on HDACs, which catalyze the amide hydrolysis of acetylated lysines. HDACs constitute a family of 18 proteins subdivided into four classes based on homology to yeast HDACs: class I (HDAC1-3, HDAC8), class IIa (HDAC4-5, HDAC7, HDAC9), class IIb (HDAC6, HDAC10), class III (SIRT1-7), and class IV (HDAC11). Alterations in their activity have been discovered in a broad range of tumors, including MM. Their targets include histones but also non-histone proteins such as p53, Hsp90, and p65 NF-κB [23].

The essential role played by HDACs in cancer and MM progression has led to the development of HDAC inhibition strategies. Pan-HDAC inhibitors seem to show stronger clinical inhibition of HDAC1, HDAC2, HDAC3, and HDAC6 than other HDACs. This suggests that their anti-tumor activity may focus on class I and class IIb HDAC inhibition [24]. Several HDAC inhibitors, such as romidespin (class I HDAC inhibitor) or panobinostat (pan-HDAC inhibitor) induce high cytotoxicity against MM cells, especially in combination with proteasome inhibitors such as bortezomib. Nevertheless, due to the wide range of targets, they also showed unfavorable side effects in clinical trials [25]. To avoid these problems, the development of selective HDAC inhibitors has become critical in MM research. To date, HDAC6 inhibitors (i.e., ricolinostat) are the ones showing encouraging results in MM treatment. HDAC6 is essential for aggresome formation, an alternative clearance pathway that is activated in response to proteasome inhibition to eliminate misfolded proteins [25]. The synergistic inhibition of proteasome and aggresome pathways leads to the accumulation of misfolded proteins, resulting in cell death [26], therefore, unveiling a promising strategy involving the combination of HDAC6 and proteasome inhibitors to tackle resistance in MM.

## 4. Non-Coding RNAs

Efforts in the study of the genome have classically focused on protein-coding genes that include only a small percentage of the mammalian genome. In the last years, a special emphasis has been placed on the non-protein-coding genome. The development of genomic and transcriptomic technologies has highlighted that 70% of the transcribed human genome corresponds to ncRNAs [27]. ncRNAs are divided in two groups: structural and regulatory ncRNAs. Structural ncRNAs include transfer RNAs (tRNAs), ribosomal RNAs (rRNAs), small nuclear RNAs (snRNAs), and small nucleolar RNAs (snoRNAs). These ncRNAs are part of the machinery involved in protein synthesis. Regulatory ncRNAs are divided depending on their size: microRNAs (miRNAs) and PIWI-interacting RNAs (piRNAs) are less than 200 nucleotides long, while long non-coding RNAs (lncRNAs) comprise the biggest. Another type of ncRNAs are circular RNAs (circRNAs), which mainly function as miRNA sponges [28].

### 4.1. microRNAs

miRNAs are 19 to 25 base-pair-long ncRNA molecules that trigger the translational repression, and sometimes degradation, of target messenger RNAs (mRNAs) with complementary sequences. Alterations in miRNAs have raised special interest in cancer research, including MM (Table 1). miRNAs constitute one of the central and most-studied post-transcriptional regulator components affecting myelomagenesis, MM progression, development, and prognosis. miRNAs can be classified into tumor-suppressive miRNAs, when they target an oncogenic gene, or oncogenic miRNAs, when they target a tumor suppressor gene, and they are tissue-specific.

**Figure 1:**
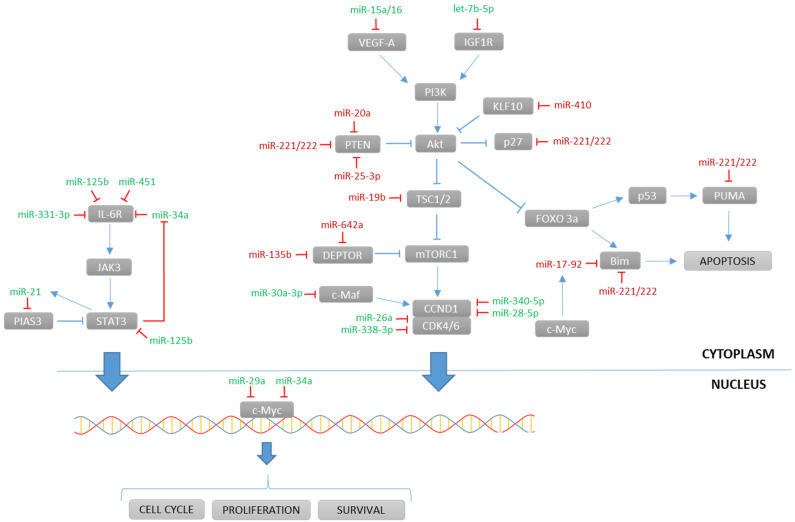
Overview of the main signaling pathways altered by miRNAs in MM cells. Oncogenic miRNAs (red) are overexpressed, and tumor-suppressive miRNAs (green) are underexpressed in MM cells.

miRNAs may act in clusters, where a group of miRNAs have their expression regulated concomitantly. One of the largest clusters involved in MM is miR-17-92, a six-member polycistronic cluster encoding for six individual miRNAs: miR-17, miR-18a, miR-19a, miR-19b, miR-20a, and miR-92a. Some of these miRNAs are known for regulating the PTEN/PI3K/Akt pathway (Figure 1). This cluster was demonstrated to take part in controlling cell proliferation, differentiation, and apoptosis, as it was positively regulated by c-Myc, which conferred to this cluster a key role in MM tumorigenesis [35]. Several studies have empirically proven, using functional assays, that *BIM* is the direct target of miR-17-92. This was confirmed in MM cells with upregulated miR-17-92 that showed an increased expression of anti-apoptotic Bcl-2 [35,78]. Despite their coordinated role, some of the miRNAs belonging to this cluster also had specific functions. Interestingly, miR-20a was highly expressed in bone marrow samples of MM patients when compared to healthy donors. The introduction of a synthetic substitutive miR-20a (mimic-based approach) showed an increased growth rate and decreased apoptosis in the U266 MM cell line, and a promoted tumor growth in a SCID/NOD mouse xenograft model [29]. *PTEN* was shown to be a downstream target of miR-20a, pointing out the PTEN/PI3K/Akt pathway as altered by miR-20a [30]. miR-19b specifically targeted the tumor-suppressive co-chaperone TSC1 and activated the mTOR pathway, which promoted cancer stem cell (CSC) proliferation [36].

The miR-221/222 tandem also contributed to MM development. Critical genes encoding for PUMA, PTEN, Bim, and p27 were described as its targets (Figure 1), and an anti-sense pharmacological strategy was useful to revert dexamethasone resistance in the MM.1S/MM.1R cellular model. Inhibition of miR-221/222 could constitute a potential therapeutic strategy for treating MM, and detection of miR-221/222 could also be useful as a diagnostic, prognostic, and therapy-assessment biomarker [33]. On another side, the miR-15a/16 tandem showed a tumor-suppressive role by repressing VEGF-A expression at a post-transcriptional level and therefore limiting tumor-induced neoangiogenesis [71].

The PI3K/Akt/mTOR pathway is one of the most affected signaling cascades in MM, and various miRNA alterations target different proteins belonging to this pathway (Figure 1). Beside miRNA clusters, single miRNAs could also disrupt the balance in the PI3K/Akt/mTOR signaling. miR-410 was found in newly diagnosed and relapsed MM patients. It was correlated to advanced ISS stages and a shorter overall survival, through targeting the zinc-finger transcription factor KFL10, which resulted in PTEN/PI3K/Akt downstream activation [34]. Alternatively, the ectopic expression of miR-135b and miR-642a decreased the expression of their target protein DEPTOR, an mTOR inhibitor which maintains plasma cell differentiation. Therefore, these two miRNAs contributed to a more dedifferentiated MM profile and a worse outcome for patients [37]. Among the tumor-suppressive miRNAs regulating the PI3K/Akt/mTOR pathway, let-7b-5p was significantly downregulated in MM tissues and RPMI 8226 cells. Its reintroduction inhibited cell growth, induced S/G2 phase cell cycle arrest, and promoted apoptosis by targeting *IGF1R* [56].

The IL-6-dependent JAK/STAT pathway is also altered in MM (Figure 1). Oncogenic miR-21 is controlled by an upstream enhancer with two strictly conserved STAT3 binding sites. Cancer cell survival induced by IL-6 and consequent activation of JAK/STAT signaling directly promoted miR-21 expression, which, at the same time, targeted the protein inhibitor of activated STAT3 (PIAS3) in a positive feedback loop [31]. This upregulation could be reversed by miR-21 inhibition. This led to reduced cell survival and proliferation in MM cell lines, as well as anti-tumor activity in NOD/SCID mice bearing human MM xenografts [79]. Tumor suppressive miRNAs, such as miR-331-3p, miR-125b, and miR-34a directly target *IL6R*, therefore conferring a strong downregulation in the JAK/STAT signaling [52,61]. 

Regulatory miRNAs are also important in cell cycle arrest through a strict control of cyclin activity. In silico target predictions suggested cyclin D1 (*CCND1*), which forms a complex with CDK4 and CDK6, as being one of the main targets of several underexpressed miRNAs found in MM patients, although experimental validation is still necessary [44]. Beyond this, miR-26a directly targeted *CDK6* (Figure 1), and its reintroduction induced cell cycle arrest at G0/G1 phase in MM cells, as well as an upregulation of p53 and p21 expression [57]. Similarly, miR-338-3p targets *CDK4*, and its overexpression induced apoptosis via caspases 3 and 8 [60]. miR-30a-3p targets c-Maf, a transcription factor, that leads to the upregulation of *CCND1*, which promotes proliferation, and increases levels of integrin β7, thus facilitating MM adhesion to the bone marrow stroma [59]. Recent studies showed that miR-340-5p and miR-28-5p directly targeted *CCND1* and these miRNAs experienced a MM-specific epigenetic silencing through promoter hypermethylation. This hypermethylation constitutes a first early step in myelomagenesis and highlights the importance of cell cycle regulation through miRNAs. Therefore, the complex interaction of miRNAs with different epigenetic factors shows their growing relevance in MM pathogenesis [58,68]. 

Proto-oncogenes such as *c-Myc* are also affected in MM. Well-defined miR-29a and miR-34a were identified to strongly inhibit cell proliferation and induce apoptosis by targeting *c-Myc* [63,65]. Mimic-based experiments suggested that a stable transfection of these downregulated miRNAs inhibited tumor growth in vitro and in MM-xenografted mice [64]. In the same way, the newly described miR-22 affected *c-Myc* signaling in vitro and restored sensitivity towards lenalidomide [62]. Various studies showed a considerable number of oncogenes that were overexpressed due to miRNA downregulation, including *MET* and *BCL2* [64,67]. 

Besides the involvement of tumor cells, it has been widely described that MM progression also critically depends on bone remodeling. miR-210 was found to be a hypoxia-inducible miRNA, which acts mainly in the bone marrow as the microenvironment becomes increasingly hypoxic. miR-210 increases the expression of factors such as CXCR4, IL-6, TGF-β, and VLA-4, leading to an inhibition of osteoblast formation. These effects highlight the key role of this regulator miRNA in microenvironment modulation [38]. Another study proposed that miR-210 could induce phenotypic changes in MM cells, going from an active normoxic anti-apoptotic phenotype characterized by IRF4 activation, to a more quiescent hypoxic and glycolysis-dependent phenotype [39]. On the contrary, miR-199a-5p plays a tumor-suppressive role by regulating a hypoxic phenotype through direct targeting of *HIF1A*, and preventing MM dissemination through DDR targeting [76]. Also, miR-342 and miR-363 exert anti-MM functions by directly targeting and inhibiting *RUNX2*, which leads to a decreased level of osteoclasts and an increased level of osteoblasts [77]. Although there is much more to elucidate in this field, these novel findings suggest that miRNA/HIF-1α could be targeted to prevent malignant progression promoted by hypoxia.

Moreover, communication between MM cells and mesenchymal stem cells (MSCs) is necessary for MM progression. Some miRNAs, such as miR-146a and miR-155, could be transferred from MM cells to MSCs through exosomes. miR-146a induced the secretion of cytokines and chemokines, including CCL-5, CXCL1, CXCL10, IL-6, IL-8, and MCP-1, resulting in enhanced MM viability and migration [42]. miR-155 also induced the expression of OCT-4 and Nanog, which maintained the stemness of MM cells [43]. Reciprocal signaling could also take place between MM cells and macrophages, as the downregulation of miR-214 in MM cells induced an increased level of CD276, leading to M2 polarization in macrophages via JAK/STAT signaling [50]. This M2 polarization is an example of immunoediting mediated through miRNAs from MM cells. The release of miRNAs through exosomes to ensure communication with the surrounding microenvironment led to their detection in the serum of MM patients. Circulating miRNAs can be used as a prognostic and diagnostic tool, as they were differentially detected in MM patients when compared to healthy donors. For instance, miR-214 and miR-135b were increased in the serum of MM patients, and their levels correlated with MM progression [45]. This was due to the release of miR-135b through exosomes by hypoxic MM cells in order to promote angiogenesis, as it targeted FIH-1 and promoted endothelial tube formation [46].

In terms of prognosis, miR-146b was found to be a biomarker in hematological malignancies such as chronic lymphocytic leukemia and acute myeloid leukemia, and it was also positively correlated with increased renal impairment, ISS value, and chromosome abnormality in MM patients [47]. Moreover, the detection of oncogenic miR-20a and miR-181a could consist of a non-invasive and fast strategy to assess prognosis. These miRNAs are known to be inhibited by bortezomib, a classical proteasome inhibitor used in MM therapy [48]. The detection of these miRNAs in the serum of MM patients treated with bortezomib could indicate the development of resistance to this drug. Apart from these already known miRNAs, wide genetic profiling studies have led to the identification of differentially expressed miRNAs with no described function in MM patients. Those miRNAs may be used as biomarkers for MM diagnosis [44]. The identification of their target could expand their therapeutic use in modulating the MM microenvironment. 

In the study of cancer epigenetics, the concept of epimiRNAs refers to the miRNAs that actively regulate epigenetic modifiers or, alternatively, are regulated by them [80]. Inside this classification, there is a high number of critical tumor-suppressive miRNAs that are silenced in MM through promotor hypermethylation [54,58,68]. More interestingly, a strong connection was found between miRNAs and Polycomb Repressive Complex 2 (PRC2). The expression of EZH2, the enzymatic subunit of PRC2, was significantly reduced in malignant plasma cells when compared to their normal counterpart, and overexpressed miR-124 inhibited its expression in MM cells [41]. In addition to this, a common H3K27/me3-marked chromatin profile mediated by EZH2 was seen in MM patients, correlating with gene silencing in advanced stages and poor clinical outcome [81]. miR-29b, an epimiRNA targeting *DNMT3* and *HDAC4*, was shown to play a key role [72]. Several studies efficiently silenced *HDAC4* through miR-29b replacement in MM cell lines, thus leading to the inhibition of cell survival and migration, as well as the induction of apoptosis and autophagy [82]. 

All these studies indicate that miRNAs play an important role in myelomagenesis and MM progression, affecting cell proliferation, neoangiogenesis, and the microenvironment, among others. This versatile and complex role of miRNAs in MM development suggests that they could be used as molecular targets to efficiently develop a therapeutic strategy, by replacement or inhibition approaches. This interaction network extends to other ncRNAs, especially lncRNA and circRNA, giving insight into new perspectives on MM molecular basis.

### 4.2. Long Non-Coding RNAs

**Figure 2:**
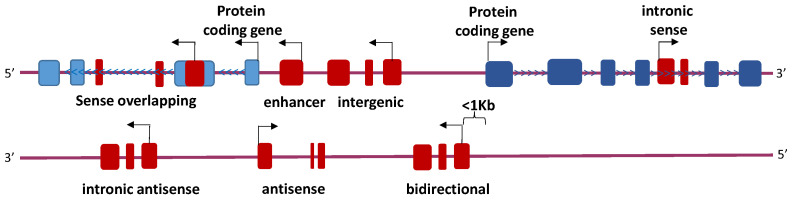
lncRNA classification attending to their localization. The enhancer, intronic, and intergenic lncRNAs contain their own promoters that are distinct from protein coding gene. Bidirectional lncRNAs share promoter with a protein coding gene and are transcribed from the opposite strand of the gene. Antisense (AS) lncRNAs have been involved in the transcriptional interference of the neighboring protein coding genes.

lncRNAs include ncRNAs whose transcripts are longer than 200 nucleotides. Their classification is performed depending on their localization (Figure 2). Nowadays, there is an incomplete understanding of the mechanism of action of lncRNAs, but it is widely accepted that they play an important role in cancer [27].

In MM, dysregulated lncRNAs affect various aspects of the disease (Table 2). Several of them act as competing endogenous RNAs (ceRNAs), having miRNAs as targets and acting as miRNA sponges (Table 3) [83].

Overexpressed ceRNAs play an oncogenic role in MM and can be possible therapeutic targets. Three lncRNAs (MALAT1, NEAT1, and UCA1), which target more than one miRNA, can be highlighted. MALAT1 targeted miR-1271-5p and miR-509-5p, which were shown to be transcriptional regulators [73,74]. At the same time, MALAT1 was the target of miR-125b, which is underexpressed in MM, showing that the inverse regulation of lncRNAs by miRNAs is also possible [51]. To date, more than 700 publications relate MALAT1 to cancer. In addition to the functions previously described, MALAT1 promoted genomic stability via the MALAT1/PARP1/LIG3 DNA repair complex in MM [89]. This lncRNA also enhanced the expression of *HMGB1*, an important gene inducing cell autophagy. Silencing MALAT1 resulted in HMGB1 degradation via ubiquitination [101] and led to the upregulation of glycolytic genes under hypoxia [102]. Moreover, MALAT1 was shown to be a biomarker of poor prognosis in MM that can be used to predict the progression of the disease [90]. Furthermore, anti-MALAT1 treatments showed a synergistic effect with PARP1 or proteasome inhibitors in vitro [89]. The other lncRNAs include NEAT1, which regulates miR-214, a prognosis biomarker, and miR-193a, which is involved in the regulation of proliferation (Table 1). Additionally, NEAT1 was linked to dexamethasone resistance [50,66], the inhibition of genes involved in DNA repair [93], and alteration of the Wnt/β-catenin signaling pathway [94]. UCA1 activated the JAK/STAT signaling pathway through downregulation of miR-331-3p, and altered transcriptional activity by targeting miR-1271-5p [61,75]. 

In general, the antisense lncRNAs are described as activity regulators for their neighboring genes. RUNX2-AS1 targets *RUNX2* pre-mRNA, the gene located on the same locus. RUNX2-AS1 was found to be elevated in MSCs derived from MM patients, and this led to a decrease in their osteogenic potential [97]. Besides, DARS-AS1 is another overexpressed lncRNA which is related to the hypoxia phenotype. In this case, DARS-AS1 did not repress the expression of *DARS* neighboring gene, but increased the expression of *RBM39* and enhanced the mTOR signaling pathway. Furthermore, DARS-AS1 may form a positive feedback loop with HIF-1α to increase the survival ability of MM cells [86]. 

HOTAIR is another lncRNA that is overexpressed in a broad spectrum of cancers, including MM, and is described as being oncogenic. It interacts with epigenetic regulators such as the PRC2 complex and lysine-specific histone demethylase 1A that regulate histone methylation in cancer tissue [103]. However, little is known about its role in MM. Guan et al. [88] described its implication in resistance to dexamethasone by mediating the JAK/STAT signaling pathway in MM cells, but more studies are necessary to elucidate the real impact of this oncogenic lncRNA in the disease.

In terms of proliferation, overexpressed PDIA3P lncRNA was described as an enhancer of the transactivation activity of c-Myc by affecting the pentose phosphate pathway signaling that plays an important role in the survival of cancer cells [96]. The upregulated lncRNAs H19, which sponges miR-29b, and SMILO are both suppressors of cell proliferation [87,98]. Other lncRNAs were described as being biomarkers of poor prognosis in MM, such as MIAT1 and NR_046683 [92,95]. Interestingly, MIAT1 is a bortezomib-inducible lncRNA and its inhibition contributed to overcome bortezomib resistance [92].

Few lncRNAs are underexpressed, such as MEG3 and BM742401, which have a hypermethylated promoter region [84,91]. Another underexpressed lncRNA worth mentioning is XLOC_013703, which regulates cell growth through the NF-κB pathway [100]. 

A recent study analyzing the lncRNA transcriptome in MM described a larger number of altered lncRNAs that could be considered as possible new therapeutic targets. This study also showed their clinical impact in the disease [98]. All of these findings highlight the need for more investigations into lncRNAs’ role, which then may lead to the improvement of treatments for MM patients.

### 4.3. Other ncRNAs

The impact of ncRNA dysregulation in MM goes beyond the well-studied miRNAs and lncRNAs. piRNAs constitute a very recent family of 24-31 nucleotide RNAs that can be abnormally expressed in various cancers. piRNA-823 is the only described example of its kind involved in MM pathogenesis so far [104]. Its overexpression was associated with a poor prognosis, suggesting that its detection could be part of a suitable risk stratification strategy. The oncogenic action of piRNA-823 seemed to be mediated through de novo methylation, as its overexpression was associated with DNMT3A/3B expression levels in primary MM cells [105]. Moreover, levels of piRNA-823 were higher in extracellular vesicles shed by MM cells, suggesting that this may promote proliferation, angiogenesis, and invasion in endothelial cells [104]. These findings reinforce the importance of cellular communication between MM cells and the microenvironment, also via piRNAs. 

snoRNAs are also relevant in cancer development. Beyond their canonical function in rRNA processing, mRNA splicing and editing, as well as stress responses, they are involved in pathological processes such as cell transformation, tumorigenesis, and metastasis. The most important finding about snoRNAs in MM involved ACA11, an orphan box H/ACA snoRNA encoded within an intron of MMSET [106]. ACA11 was found to be localized into nucleoli and bound to a small nucleolar ribonucleoprotein (snRNP). This led to the downregulation of ribosomal protein genes that are associated with the control of oxidative stress [107]. Recently, new studies showed that ACA11 upregulated ribosome biogenesis in a reactive oxygen species-dependent manner, suggesting that the increased level of protein synthesis driven by ACA11 made MM cells more sensitive to proteasome inhibitors [108]. Moreover, elevated levels of tRNA were seen in MM cells to accommodate their increased need for protein translation machinery [109]. Therefore, it is reasonable to state that the detection of this snoRNA could help assess the efficacy of a bortezomib-based therapy.

Recently, circRNAs has been seen as a promising new therapeutic approach for MM. circRNAs are covalently-closed RNAs due to the junction of their 5′ and 3′ ends, which can remain relatively stable in the cytoplasm. This closed structure confers them an important variety of functions, such as acting as miRNA sponges, interacting with RNA binding proteins, or acting as scaffolds for the formation of enzyme-substrate complexes. circRNAs were identified as being key regulators of some hallmarks of cancer, including unaltered growth, apoptosis evasion, limitless replicative potential, sustained angiogenesis, tissue invasion and metastasis, as well as stemness [28]. A recent analysis of the genome wide profiling showed circRNA expression patterns in MM [110]. circ_0000190 was found to negatively regulate miR-767-5p in the cytoplasm and to inhibit cell viability, proliferation, and MM progression in both in vitro and in vivo models through the MAPK4 pathway [68]. Besides, circ-CDYL was found to regulate miR-1180 and to overexpress *YAP*, ultimately triggering MM uncontrolled growth [70]. The duality of functions between suppressive and inductive tumor roles is also present in circRNAs and some were proposed as possible biomarkers. hsa_circ_0007841 was upregulated in MM cell lines, but also differentially expressed in MM patients depending on their staging. Besides, it targeted several miRNAs regulating bortezomib sensitivity and osteoclast differentiation [111]. The newly described hsa_circ_0003489 induced the overexpression of HDAC1 by sponging its repressor, miR-874-3p, and maintaining cell viability and proliferation. Its knock-down led to a sensitivity to bortezomib [69]. These new findings reinforce the idea that circRNAs could be suitable molecular targets in innovative therapies against MM and that their detection could be valuable for assessing and monitoring MM development in patients.

## 5. Therapies

The treatment options for MM patients are based on the use of a combination of various drugs. They can be classified as targeted therapies (proteasome inhibitors, HDAC inhibitors, monoclonal antibodies, and immunomodulatory agents), chemotherapeutic agents, and immunotherapy. The treatment of MM can also include an autologous stem-cell transplantation, taking into account multiple factors as described by Palumbo et al. [112]. However, the main problem with these therapies is that most patients develop relapse or become refractory to treatments. Also, side effects must be kept in mind [2]. Therefore, different therapeutic strategies are required to improve the outcomes of MM patients. One direction could be by modulating classical epigenetic mechanisms, such as methylation and acetylation. In terms of methylation, DNMTi, such as 5-azacytadine and decitabine, were used to restore the methylation pattern and offered promising results, encouraging the development of new demethylation agents. Nevertheless, most efforts are currently devoted to modulate acetylation with the use of HDAC inhibitors. The introduction of the pan-HDAC inhibitor panobinostat into the clinic provided significant efficacy but also several side effects were observed. Among HDAC inhibitors, HDAC6-selective inhibitors are also showing clinical value with an improved safety profile [31]. These findings highlight HDAC6 as one of the main promising targets in seeking new anti-MM agents.

## 6. Future Perspectives

Recent findings on dysregulated ncRNA have pointed out the possibility to target them in MM. The identification of ncRNAs playing key roles in MM development suggested that the introduction of mimic RNAs or inhibitors could constitute a next step in MM therapy. In order to optimize these therapies, important efforts are being put in the development of delivery strategies, such as the use of tissue-specific or cancer-specific promoters, adenoviruses, adeno-associated viruses, and lentiviruses [113].

An important number of altered miRNAs that dysregulate crucial signaling pathways such as PI3K/Akt and JAK/STAT were found in MM (Figure 1). The majority of those miRNAs have identified targets, suggesting that their modulation could be used as a therapeutic strategy. Various in vitro and in vivo studies showed that the inhibition or restoration of altered miRNAs could exert antiproliferative activity [56,79]. Therefore, more efforts are needed to translate these possible new miRNA-based therapeutic strategies to MM patients. Combination therapies targeting various miRNAs added to classical therapies such as bortezomib or HDAC inhibitors could be another possible approach to offer synergistic effects. These combination therapies could also include the use of lncRNAs. Their number is growing with the use of recent advances in sequencing technologies. They exert transcriptional and regulatory functions that are so far not well-described. The considerable number of altered lncRNAs found in MM opens the door to many therapeutic opportunities that should be investigated, as lncRNAs have been reported to directly target genes and miRNAs. Furthermore, there is a growing interest towards circRNAs. This new group of ncRNAs has already shown gene regulatory potential, due to their miRNA sponge action [28]. Their implication in MM development suggests that circRNA-based therapy could bring important benefits, and circRNAs have become attractive thanks to their versatile interaction with multiple biomolecules beyond miRNAs. It reinforces the idea that an eventual circRNA-based therapy in MM could lead to strong anticancer effects. However, there is a need for more research to expand the number of identified circRNAs contributing to MM development.

Alternatively, the CRISPR-Cas9 technology is a new tool that has emerged as an exciting new approach to cancer therapy. Although, initially, the CRISPR-Cas9 technique raised special controversy due to off-target events, much progress has been made in this regard and the advantages of this technology cannot be ignored. The ability to target specific genes gives the opportunity to knockdown overexpressed genes and can be used to obtain synergy by targeting multiple genes [114]. The ncRNAs or epigenetic markers that disrupt normal gene expression may therefore be suitable targets. In the case of MM, the possibilities of therapy are important due to the high number of dysregulated elements previously discussed. For example, a combined targeting of miR-19a and MALAT1 may cause synergistic effects, since both are overexpressed and play key roles in MM progression. 

Moreover, when a lncRNA regulates a miRNA (Table 3), a loop can be created by using a “miRNA-responsive CRISPR-Cas9 switch”, in which the expression of Cas9 is modulated depending on miRNA levels [115]. For example, MALAT1 has been shown to inhibit the expression of miR-1271-5p, which led to an increase in cell viability and invasion. If MALAT1 levels are high, miR-1271-5p levels decrease and Cas9 starts editing MALAT1. When MALAT1 is low, miR-1271-5p increases again and blocks Cas9 activity. This innovative mechanism can help regulate the expression of oncogenic lncRNAs that are controlled by miRNAs. 

Nuclease-dead Cas9 (dCas9) fusion proteins have been specifically designed for guiding modifiers to modulate epigenetic markers, as well as the chromatin state, depending on the elements fused to them [116]. For instance, epigenetic modifiers, such as p300 or histone-modifying enzymes (e.g., DNMT3A and HDAC3), induced corrections in the epigenetic pattern [117,118,119], which led to the reverting of alterations found in MM. Recently, the CRISPR-Cas13 system was developed to target RNA, providing a useful new tool that can be applied to target altered ncRNAs [120]. These are possible uses of the CRISPR-Cas technology, but the field is rapidly evolving, and new approaches should be regularly evaluated.

## 7. Conclusions

Recent findings on epigenetic and ncRNA alterations involved in MM have suggested their importance in the development and progression of the disease. A considerable amount of these dysregulations affects crucial pathways implicated in the cell cycle, proliferation, genomic stability, angiogenesis, and hypoxia. Besides, the identification of ncRNA biomarkers suggests their potential use as efficient diagnosis and prognosis tools. However, more research is still required. The emergence of techniques such as CRISPR-Cas opens the way for possibilities to develop new treatments and improve the outcome of MM patients.

## Figures and Tables

**Table 1:** miRNAs dysregulated in MM.

Activity/Pathway Affected	miRNA	Status ^1^	Target	References
Enhances PI3K/Akt pathway	miR-20a	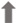	*EGR2*, *PTEN*	[29,30]
	miR-21	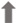	*PIAS3*	[31]
	miR-25-3p	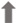	*PTEN*	[32]
	miR-221/222	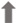	*PUMA*, *PTEN*, *CDKN1B*, p27	[33]
	miR-410	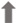	*KLF10*	[34]
Enhances mTOR pathway	miR-19b	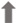	*TSC1*	[35,36]
	miR-135b, miR-642a	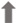	*DEPTOR*	[37]
Related to a hypoxia phenotype	miR-210	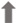	*DIMT1*	[38,39]
	miR-1305	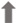	*MDM2*, *IGF1*, *FGF2*	[40]
Disrupts PRC2 activity	miR-124	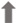	*EZH2*	[41]
Modulates microenvironment	miR-146a	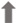	Not described	[42]
	miR-155	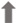	Not described	[43]
Promotes proliferation, circulating miRNAs	miR-17-92	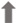	*BIM*	[35]
	miR-221/222	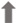		[33]
Circulating miRNA	miR-1	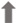	Not described	[44]
	miR-133a/b	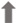	Not described	[44]
	miR-135b	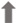	*HIF1A*	[45,46]
	miR-146b	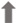	Not described	[47]
	miR-181a	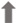	*BCL2L11*	[48,49]
	miR-214	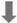	*CD276*	[50]
Represses JAK/STAT pathway	miR-125b	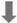	*IL6R*, *STAT3*, *MALAT1*	[51,52]
	miR-331-3p	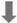	*IL6R*	[53]
	miR-375	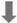	*PDPK1*	[54]
	miR-451	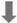	*IL6R*	[55]
	let-7b-5p	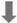	*IGF1R*	[56]
Regulates cyclin activity	miR-26a	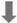	*CDK6*	[57]
	miR-28-5p	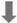	*CCND1*	[58]
	miR-30a-3p	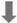	*MAF*	[59]
	miR-338-3p	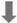	*CDK4*	[60]
	miR-340-5p	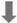	*CCND1*, *NRAS*	[61]
	miR-196a/b	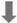	*CCND2*	[44]
Regulates proliferation	miR-22	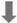	*c-Myc*	[62]
	miR-29a	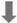	*c-Myc*	[63]
	miR-34a	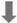	*BCL2*, *CDK6*, *NOTCH1*, *c-Myc*, *MET*, *IL6R*	[52,64,65]
	miR-193a	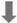	*MCL1*	[66]
	miR-497	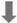	*BCL2*	[67]
	miR-767-5p	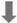	*MAPK4*	[68]
	miR-874-3p	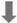	*HDAC1*	[69]
	miR-1180	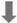	*YAP*	[70]
Prevents angiogenesis	miR-15a/16	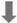	*BCL2*, *VEGF*, *IL17*	[71]
Regulates acetylation	miR-29b	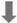	*HDAC4*, *MCL1*	[72]
Regulates transcriptional activity	miR-509-5p	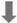	*FOXP1*	[73]
	miR-1271-5p	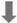	*SOX13*, *HGF*	[74,75]
Prevents hypoxia phenotype	miR-199a-5p	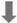	*HIF1A*, *VEGFA*	[76]
Prevents osteolytic activity	miR-342	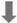	*RUNX2*	[77]
	miR-363	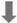	*RUNX2*	[77]

^1^ Arrow up indicates overexpression of the miRNA, and arrow down indicates underexpression of the miRNA.

**Table 2:** lncRNAs that are dysregulated in MM.

lncRNA	Status ^1^	Target	Activity/Pathway Affected	References
ANGPLT1-3	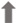	miR-30a-3p	ceRNA	[59]
BM742401	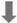	Not described	Inhibit myeloma cell migration, biomarker	[84]
CRNDE	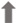	miR-451	ceRNA	[55,85]
DARS-AS1	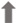	*RBM39*	Enhances mTOR pathway, hypoxia phenotype	[86]
H19	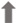	miR-29b	ceRNA, biomarker	[87]
HOTAIR	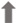	Not described	Enhances JAK/STAT pathway	[88]
MALAT1	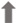	*HMGB1*, miR-509-5p, miR-1271	Contributes to genomic stability, ceRNA, biomarker	[73,74,89,90]
MEG3	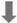	miR-181a	Promotes osteogenic differentiation, biomarker, ceRNA	[91]
MIAT	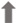	miR-29b	Inducible by bortezomib, ceRNA, biomarker	[92]
NEAT1	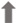	miR-214, miR-193a	Downregulates genes involved in DNA repair, enhances Wnt/β-catenin pathway, ceRNA	[50,66,93,94]
NR_046683	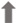	Not described	Biomarker	[95]
OPI5-AS1	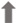	miR-410	ceRNA	[34]
PDIA3P	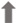	*c-Myc*	Regulates proliferation	[96]
RUNX2-AS1	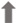	*RUNX2* pre-mRNA	Promotes osteogenesis	[97]
SMILO	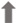	Not described	Regulates proliferation	[98]
SNHG16	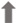	miR-342	ceRNA	[99]
UCA1	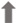	miR-1271-5p, miR-331-3p	ceRNA	[61,75]
XLOC_013703	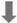	*IKKA*	Represses NF-κB pathway	[100]

^1^ Arrow up indicates overexpression of the lncRNA, and arrow down indicates underexpression of the lncRNA.

**Table 3:** Genes dysregulated in MM due to overexpression of ceRNA lncRNAs.

lncRNA	miRNA	Gene	References
ANGPLT1-3	miR-30a-3p	*MAF*	[59]
CRNDE	miR-451	*IL6R*	[55,85]
H19	miR-29b	*HDAC4* and *MCL1*	[72,87]
MALAT1	miR-509-5p	*FOXP1*	[73]
	miR-1271-5p	*SOX13*	[74]
MEG3	miR-181a	*BCL2L11*	[91]
MIAT	miR-29b	*HDAC4* and *MCL1*	[72,92]
NEAT1	miR-214	*CD276*	[50]
	miR-193a	*MCL1*	[66]
OPI5-AS1	miR-410	*KLF10*	[34]
PRAL	miR-210	*DIMT1*	[38,39]
SNHG16	miR-342	*RUNX2*	[99]
UCA1	miR-331-3p	*IL6R*	[61]
	miR-1271-5p	*SOX13* and *HGF*	[75]
